# The Latin America Early Career Earth System Scientist Network (LAECESS): addressing present and future challenges of the upcoming generations of scientists in the region

**DOI:** 10.1038/s41612-022-00300-3

**Published:** 2022-10-20

**Authors:** Ana María Yáñez-Serrano, Maricar Aguilos, Cybelli Barbosa, Tomás Rafael Bolaño-Ortiz, Samara Carbone, Stephanie Díaz-López, Sebastián Diez, Pamela Dominutti, Vanessa Engelhardt, Eliane Gomes Alves, Jenniffer Pedraza, Jorge Saturno, Zitely A. Tzompa-Sosa

**Affiliations:** 1 IDAEA-CSIC, Barcelona, Spain; 2 CREAF, Bellaterra (Cerdanyola del Vallès), Catalonia, Spain; 3 Global Ecology Unit, CREAF‐CSIC‐UAB, Catalonia, Spain; 4 Department of Forestry and Environmental Resources, North Carolina State University, Raleigh, NC USA; 5 National Institute for Amazonian Research, Manaus, Brazil; 6 Centre for Environmental Technologies, Universidad Técnica Federico Santa María (CETAM-USM), Valparaíso, Chile; 7 Federal University of Uberlandia, Agrarian Sciences Institute, Santa Mônica, Uberlândia - MG Brasil; 8 Centro de Ciencias Atmosféricas y Biogeoquímica, Instituto Venezolano de Investigaciones Científicas, Caracas, Venezuela; 9 Wolfson Atmospheric Chemistry Laboratories, University of York, Innovation Way, Heslington, York UK; 10 Universidad Tecnológica Nacional, Còrdoba, Argentina; 11 University Grenoble Alpes, IRD, CNRS, Grenoble, France; 12 Leibniz Institute for Tropospheric Research, Leipzig, Germany; 13 Department of Biogeochemical Processes, Max Planck Institute for Biogeochemistry, Jena, Germany; 14 Department of Climate and Environment, National Institute of Amazonian Research, Manaus, Brazil; 15 Universidad Nacional de Colombia, Bogotá, Colombia; 16 Physikalisch-Technische Bundesanstalt, Braunschweig, Germany; 17 Laboratoire des Sciences du Climat et de l’Environnement, Gif-sur-Yvette, France

**Keywords:** Education, History, Environmental impact

## Abstract

Early career (EC) Earth system scientists in the Latin America and the Caribbean region (LAC) have been facing several issues, such as limited funding opportunities, substandard scientific facilities, lack of security of tenure, and unrepresented groups equality issues. On top of this, the worsening regional environmental and climatic crises call for the need for this new generation of scientists to help to tackle these crises by increasing public awareness and research. Realizing the need to converge and step up in making a collective action to be a part of the solution, the Latin America Early Career Earth System Scientist Network (LAECESS) was created in 2016. LAECESS’s primary goals are to promote regional networking, foster integrated and interdisciplinary science, organize soft skills courses and workshops, and empower Latin American EC researchers. This article is an initial step towards letting the global science community grasp the current situation and hear the early career LAC science community’s perspectives. The paper also presents a series of future steps needed for better scientific and social development in the LAC region.

## Present regional scientific challenges in LAC

### Regional environmental climatic issues and their consequences in LAC

The impacts of climate change in the LAC region are observed in the atmosphere/hydroclimate, oceans, and cryosphere which directly impact the biosphere. They have been identified and quantified as altered precipitation regimes, higher atmospheric and sea-surface temperatures, higher risks of droughts, increasing aridity, glacier mass loss, relative sea-level rise, higher intensity of tropical cyclones, marine heatwaves and significant loss in biodiversity^1–3^. More specifically, LAC contributes 10% of the global CO_2_e emissions, where land use and change forestry is the major contributor^4^. This is mainly related to deforestation and forest fires due to agricultural, livestock and urbanization expansion processes, especially in the Amazon Forest^5^. In Central America significant warming trends between 0.2 °C and 0.3 °C per decade have been observed in the last 30 years. Long-term observed precipitation trends show an increase over south-eastern South America and a decrease in most tropical land regions. On the Atlantic coast of South America, the rate of sea-level rise is higher than the global mean (~3.6 mm yr^−1^) and is lower along the Pacific coast (2.94 mm yr^−1^). Similar results were observed on the Caribbean Sea/Gulf of Mexico side (3.7 mm yr^−1^) and the Pacific side (2.6 mm yr^−1^)^3^. CO_2_ absorption by the seawater also results in lower pH values, which is known as ocean acidification. In South America, ocean acidification is affecting the Humboldt Current, one of the world’s four major upwelling systems, provoking negative impacts on key ecosystems^6^. Moreover, recent studies showed that the rate of glacial mass loss in the entire Andes Mountains amounted to −0.72 ± 0.22 meters of water equivalent y^−1^ from 2000 to April 2018^7^. As the Andes glacier volume loss and permafrost thawing increase, there will be significant reductions in river flow and potentially high-magnitude glacial lake outburst floods^8^. The coupling between Earth-Climate systems should also be considered, for example: it has been demonstrated that the Amazonian biomass burning reaches the Andean glaciers, which accelerate its melting^9^, and consequently raises sea-levels. This directly affects coastal areas like the Caribbean, Guatemala, Colombia, Venezuela, Brazil, Uruguay, Argentina, among others. All of these changes result in biodiversity losses^4,10^. In LAC, biodiversity hotspots and areas of high endemism are abundant. The LAC region contains about 60% of the global terrestrial life and diverse freshwater and marine species, 12% of the world’s mangrove forests, the most extensive wetlands, and 10% of the world’s coral reefs. However, there has been a decline in species abundance and a high risk of species extinction^11,12^.

When combined and extended into the economic and social dimensions, all these impacts provide excellent conditions to exacerbate socio-economic distortions and inequalities in the LAC region^1,13^. The socio-economic conditions of the region, characterized by poverty and high inequality, increased the vulnerability to these scenarios^14^. For example, considering the importance of the agricultural sector in LAC in producing food and ecosystem services for the entire planet^15,16^, the climate change impacts will affect food production, processing, storage, transport, and distribution, which could impoverish the regional economies^1,15,17^. In addition, populations located in the remote areas of LAC, indigenous people, and communities dependent on agricultural or coastal livelihoods will be especially at risk^18,19^. Additionally, climate change increases vector and viral diseases’ development and spread, resulting in worldwide consequences^20–22^.

### General public information and perception about the environmental and climatic impacts

General public information and perception are essential to combat climate change, not only by changing their lifestyles but also by voting for decision-makers that can promote environmental protection policies. In the past five years, there has been increasing awareness (from 56 to 67%) of climate change as a threat^23^. Eighteen LAC countries highlight the importance of climate change for the population. On average, 83% of the people in these eighteen countries agree that anthropogenic activities are the leading cause of the impacts, and 70% agree that mitigation and adaptation should be a priority^24^. Most individuals in LAC see the issue of climate change as a serious threat^25^. This can result from local communities witnessing regional impacts, as described in Section 1.1. This awareness is dependent on the regional historical context and level of education^24^. Education is one of the main drivers of public engagement. However, in recent years the rise of the scientific scepticism movement as part of climate change denial is one of the reasons why science is not recognized as important by both the public and the governments^24^. A recent study by Soh (2019)^26^ notes that in LAC the relative distancing of the general public from the benefits of scientific discovery has made science and technology funding an easy political target for fiscal restraint in an economic downturn. Therefore, EC scientists have had to change their approaches to reach the public and change the perception of environmental and climatic issues. By enhancing EC networks like LAECESS, these new approaches can be addressed collectively.

Although the local political context does not promote an environment to debate and shape new attitudes^25^, the increasing perception of the severity of climate impacts needs to be aligned with concrete actions to minimize climate change impacts. Mitigation strategies can improve energy generation, urban transport, agriculture, food distribution, and population health with public support. The World Economic Forum^27^ highlighted the focus on expanded investments in research and innovation to allow new markets for the future, embracing diversity and inclusion. Thus, science should be transparent, easy to understand, and integrated into life.

### LAC early career challenges

While many Latin America and Caribbean (LAC) region researchers aim for a high level of research, they are constrained due to lack of funding support, limited access to grant opportunities, substandard laboratory facilities and equipment, low salaries, and lack of tenure security^28^. From 2010 to 2019 the number of students enrolled in higher education programs increased by 38%, while the amount of money spent for the same period was, on average, 1.3% of the gross domestic product^29^. Of the total number of graduates during 2019, 35.7% were from Education, Natural sciences, Technology, and Engineering, while 64.3% studied Arts, Humanities, Business, and Welfare courses related, considering the entire LAC^29^. Argentina and Puerto Rico are the countries with a higher percentage of graduates in the Natural science field.

There are not enough research positions in LAC scientific institutions to absorb outstanding investigators. According to the International Labour Organization, millions of formal working positions were lost in Latin America during the last four years, with an impact on women and youth^30^. The employment rate recovery occurs for informal jobs (i.e., no official contract, few or absent benefits, no insurance, irregular working hours, non-paid extra hours). In turn, brain drain takes place^31^. This type of migration, where highly skilled professionals leave their home countries seeking for better working conditions just increased over the years^32^. In average, 10 to 40% of the LAC brains went to the U.S. over the last five years^33^. Many researchers, especially EC scientists, decide to leave the region, looking for proper venues and adequate support^34^. Researchers find more freedom, stability, resources, and grants to implement their scientific endeavours in many developed countries abroad, especially if they have the chance to perform study or training during master’s or Ph.D. degree^35^.

**Fig. 1:**
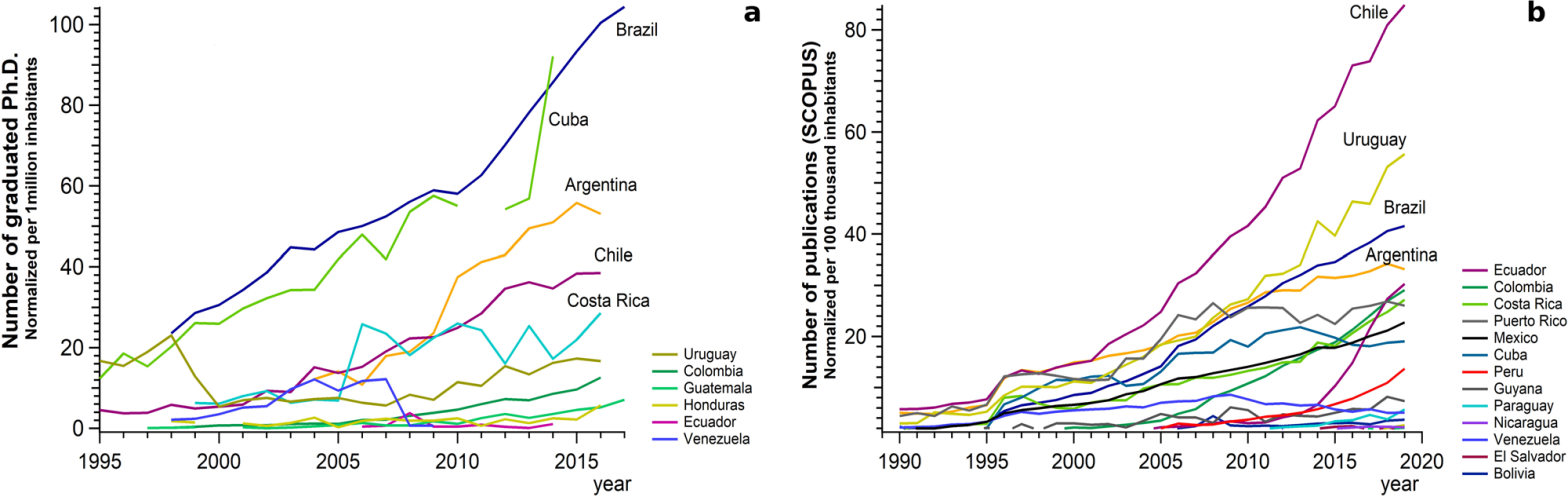
Comparison, per country, of PhD graduations and publications over the last 3 decades. Number of graduated Ph.D. in time normalized per 1 million inhabitants in **a** and number of publications in SCOPUS in time normalized per 1 million inhabitants in **b**. Data are accessible at Red RICYT^37^.

During the 2000’s, a dynamic internal movement was observed with the increase of intraregional educational policies, regional mobility programs and governmental scholarships for higher education/training and work exchange within LAC, but the numbers have been decreasing since 2017^36^. The number of graduates in LAC increased by 42% in one decade^29^, with the highest numbers from Chile, Uruguay, Brazil, and Argentina, respectively (when data normalized per 1 million inhabitants)^29,37^ (Fig. 1a). The number of publications followed the same tendency by considering the American continent (Fig. 1b). Over the last century, the overall publications on the general climate topic were primarily done in the U.S. (83%), with only 17% of the publications spread over LAC^38^. The total number of scientists in LAC is six times lower than in U.S.^39^. The low scientific productivity in the LAC region is not the result of a lack of excellence or creativity. Instead, it reflects the absence of a long-term scientific policy that is a common factor in most LAC nations^28^. LAC earth system science EC researchers are vital for tackling the regional issues previously mentioned, as they have the expertise of these issues with a global perspective combined with a cultural background that allows the applicability of a worldwide scientific issue to the regional and local context^35,40,41^. For example, previous experiences in Africa^42,43^ showed that research done without local expertise might exclude crucial local knowledge, not be locally relevant or applicable, or miss local-based solutions of potential global importance.

Due to the immediate impacts caused by climate change in most areas in LAC, in this paper, we call for a comprehensive research system and more significant support for the EC of LAC researchers, which is crucial to ensure more robust scientific research and clear career paths for EC researchers.

### Equality issues of underrepresented groups within the Latin American and Caribbean scientists

Historically, Science, Technology, Engineering, and Mathematics (STEM) has been traditionally patriarchal and has cultivated a false assumption that men are innately well-suited for scientific research while women are not^44^. However, many studies have been conducted to disprove this idea and encourage gender equality policies to correct the imbalance between men and women in science^45^. Despite all these efforts, a considerable challenge for implementing equitable policies is the lack of information on the extent and magnitude of gender imbalance in science in nations with low research and development expenditure^46^.

According to the Scimago Country Rankings, most of the scientific publications authored by researchers in the LAC region from 1996 to 2020 were produced by five countries - Brazil, Mexico, Argentina, Chile, and Colombia, covering 88.26% of all scientific topics and 86.56% of environmental science^47^. It is important to note that these are total numbers for scientific publications and are not separated by gender. If gender disparity is considered among these five countries, only Argentina and Brazil have achieved gender parity in the number of women employed in research^48,49^. The ranking suggests that inequalities might be worse in countries where science suffers from a substantial lack of funding^50^, which might prevent the creation of programs that stimulate the increase of women in science.

In 2019, the UNESCO Institute for Statistics (UIS) presented data on the number of women employed in research and development. Although the rate of employed women in the LAC region (45.1%) represented relatively good performance in worldwide indexes of gender parity in science, especially when compared to the global rate (29.3%), women researchers in LAC still face many challenges as discrimination, unequal pay and funding disparities when pursuing a career in science^46,48,51^. In general, the UIS numbers are based on headcounts, the total number of women employed in research and development, and therefore do not differentiate STEM from other sciences with higher women representativeness (e.g., humanities and social sciences^51^). Additionally, the total numbers lack an explanation for the larger gender disparities in senior levels, the large gap in authorship positions associated with seniority, the fewer women authors in prestigious journals, and the higher number of men to be invited by journals for paper submission^52^.

Other groups are underrepresented in STEM in LAC institutions. The LGTBIQA+ group is one of them. However, we have not found studies that directly tackled the representativeness of LGTBIQA+ within STEM in LAC. Our searches brought to us blogs and organizations (e.g., the STEM village; https://www.thestemvillage.com) that offer a platform for improving LGTBIQA+ visibility, but it has not been found statistics that could provide a better perspective of this community within LAC scientists. In addition, other groups such as Black people, indigenous, and *mestizos* face consequences of inequalities within STEM in LAC. The racialized structure of STEM higher education perpetuates enormous disparities resulting from structural racism, which informs and is reinforced by discriminatory beliefs, policies, values, and resource distribution^53^. In LAC, in countries like Mexico and Brazil, *mestizaje*, or racial and cultural mixing, were projects advertised as the symbol of the nation and the hope of its future to offer an idea of high tolerance in LAC; however, behind this idea, the *mestizaje* had a role by encouraging mixing to further whitening, by denying Black and indigenous identities and cultures, and by homogenizing the racial and ethnic distinctions necessary for anti-racist mobilization^54^. These historical facts have certainly impacted the under representativeness of these groups within STEM in LAC, but the lack of studies prevents us from showing statistics for the whole LAC. This presents an acute symptom of the reality in LAC, in a sense that discussions on the representativeness of Black people, indigenous, and *mestizos* are still very preliminary within STEM in LAC and that scientific research on this topic is probably concentrated in humanities and social sciences.

Recently, the socio-economic crisis imposed by the COVID-19 pandemic has intensified the problems faced by underrepresented groups in LAC. Gender, race, parenthood, and intersectionality are essential factors in assessing how the pandemic has affected scientists. Recent studies have shown that women and mothers are the groups taking the strongest hit in academia in the US and Europe, with lower rates of paper submission and publication, as well as fewer grant proposal submissions during the pandemic^55–58^. In LAC, we found similar results in a comprehensive study conducted in Brazil that revealed that in STEM, male academics (especially those without children) were the least affected, whereas Black women and mothers were the most impacted scientists^49^. These impacts are likely a consequence of the exacerbated unequal division of domestic labor during the pandemic, and that racism strongly persists in academia, especially against Black women; in addition, EC scientist women (regardless of race) might have been disproportionately affected since the early career period aligns with the reproductive age of these women^58^. Young children require much more attention and care, which was exacerbated during the period of social isolation. That can reduce the number of hours dedicated to research and likely to paper submission^56–58^. These recent studies suggest that the pandemic will have long-term effects on the career progression of these already underrepresented groups and that affirmative policies are urgently needed for reparation.

In summary, despite recent progress, the gender disparity in LAC science is likely to persist for generations. Gender imbalance in the LAC region is deeply affected by Latino cultural stereotypes, notably contributing to Latinas leaving academia^59^. Therefore, the combination of limited funding and poor working conditions promotes a reduction of women in science and can create a collateral brain drain, with Latinas moving to high-income or less-unequal countries to pursue a career in science. The contribution of other underrepresented groups—LGTBIQA+, Black people, indigenous, *mestizos*—within STEM in LAC and their intersectionality need to be further investigated to offer a deeper view of the current situation. Within the LAECESS network (see next section), we expect to boost discussions on how to increase the number of underrepresented groups in science, especially in Earth system sciences, including discussions on promoting their access to seniority positions within their countries. For the LAECESS network, it is imperative to increase the number of underrepresented groups as diversity is a keystone for building high-quality and innovative science^60,61^.

## LAECESS development

The creation of the Latin America Early Career Earth System Science Network (LAECESS) was motivated by bringing ECs together, joining the members of two Latin American EC Groups: iLEAPS (integrated Land Ecosystem—Atmosphere Processes Study)^62^ and IGAC (International Global Atmospheric Chemistry)^63^. These two networks, hosting conferences every two years and plenty of webinars during the year, are global networks. iLEAPS has regional ECS groups whose goal is to provide opportunities to meet and engage with their interdisciplinary international broad community, where ECS can connect with established scientists through workshops, training events, and regional networks. On the other hand, IGAC has regional networks, such as IGAC Americas Working Group (although not ECS only), which goal is to contribute to the development of a scientific community focused on building collective knowledge in/for the Americas including training and fostering the next generation of scientists^64^. Both groups often meet at conferences, and some members were or are part of both networks. In this sense, LAECESS founders felt the need to widen and join these two LAC ECs groups into a broader network. The networks above are still active, and LAECESS serves as a bridge between both ECS communities.

Since its foundation in 2016, LAECESS has been an open platform for Earth system scientists with origin and/or research interests in Latin America to connect and share knowledge. LAECESS’s primary goals are to promote regional networking, foster integrated and interdisciplinary science, organize soft skills courses and workshops, and empower Latin American EC researchers.

The LAECESS network has grown over time and currently holds 115 young scientists, including 25 on its steering committee. Over its six years of existence, LAECESS has organized multiple activities, including presentations at conferences, webinars, EC breakers in conferences and workshops (such as the early career event at the Land- atmosphere workshop held June 10–11^th^ 2021^65^), periodic blog posts, where members share career advice, views on scientific topics with a communication outreach approach (climate change, air quality, health, COVID-19 pandemic, Amazonian deforestation, etc.) and brief reports explaining our research to a broader audience. Additionally, LAECESS currently has social media accounts and hosts a permanent website, all interconnected and moderated by different steering committee members who frequently communicate with each other. This cooperative way of managing our social media brings different views and discussions to the table, always empowering and respecting our diversity of thoughts, cultures, and scientific limitations. Moreover, we use a mailing list to share job offers, funding opportunities, scientific studies, research questions, and a biannual newsletter.

Additionally, collaborations among LAECESS members are catalyzing collaborations and joint scientific publications^14,66^. In July 2021, LAECESS offered its first webinar entitled: *“Past, present and future of Earth System Sciences,”* which had over 50 early career participants and presented senior keynote speakers from the LAC region. Overall, these activities help promote scientific development in LAC by enabling LAC EC with free online tools. Another example is the start of a database containing information on LAC scientists putting at the disposal of LAECESs community available resources, tools, and information. We are currently working on this.

One key aspect of LAECESS is the networking diversity of its members who at the same time, are members of other important networks, enhancing LAECESS visibility and outreach. These networks are YESS (Young Earth System Scientists Community)^67^, FutureEarth, ESWN (Earth System Women Network)^68^, ILEAPS^62^, IGAC^63^, Fluxnet, Ameriflux, APECS (Association of Polar Early Career Scientists) or the Polar Science Early Career Community Office (PSECCO). Additionally the reason for not merging with other networks is the scope of the networks. LAECESS has a LAC-based scope, different from other networks. For instance, the network YESS (Young Earth System Scientists Community), which has a more worldwide scope, is an interactional EC network in climate and meteorology closely related to the World Meteorological Organization^67^. On the other hand, the networks APECS or PSECCO are very specific to the cryosphere. Nevertheless, we have LAECESS members in many networks who actively collaborate and have promoted internal communication regarding events, opportunities, and future projects.

After six years since its origin, LAECESS’s has become an active early career network. No matter where our members go, they keep strengthening our network, creating links with each other, and promoting the LAECESS goals. LAECESS’s biggest asset is its membership because even though members live in almost every time zone, we remain connected. LAECESS continues to invite everyone, regardless of gender, ethnicity, color, and origin. Our collective effort is our strength.

## Future steps

LAECESS has identified priority actions to improve the LAC region’s scientific environment during these years. particularly for early careers. Namely, these are network sustainability over time, the need for a regional open-access information repository, improvement in scientific communication, early career involvement in decision-making, improved research funding opportunities, collaboration with other networks and initiatives, and increased representation for women in LAC earth science careers.

### Network sustainability

Given the early career nature of LAECESS, as for any other early career network, there is a continuous flow of people in the network, and for it to be sustainable over time, a clear strategy must be established, such as mentoring activities from former chairs to new ones. In addition, communication among network peers is critical. In this regard, a monitoring plan is necessary always to include and engage incoming members. Some tools for achieving this purpose would be: (a) a clear description of the network’s purposes and expressing the need for this network for the Latin American Earth System Science community, and (b) training activities and an organizational structure for new members to follow.

### Regional open-access repository

The scientific LAC community would greatly benefit from public access to journal collections and data repositories. Some effort has been made in such regard with the SciELO initiative^69^. However, future and current challenges for earth system scientists in the region will require strong collaboration among research groups. LAECESS proposes an electronic LAC platform where the research groups could be listed and georeferenced. This platform would contain basic information about each group and observatory stations and modeling tools (including open data), and researchers can easily contact one another. This approach would significantly enhance LAC region scientific collaborations and consequent development. The change of research workflows and practices toward Open Science approaches requires efforts beyond the individual initiative. Universities and research institutions are required to design Open Science strategies and to implement the necessary technology to improve the transition.

### Improvement in scientific communication

Scientific communication is essential for generating impactful results and social engagement. EC scientists would greatly benefit from tools and techniques for effective communication. Scientists should be able to attractively engage with the general public by understanding the broad implications of their research, understanding their audience, and adapting their speech for non-technical audiences. One crucial step to improve scientific communication would be hiring scientific journalists to assist in science dissemination and outreach. Scientific journalists can also help train the LAC research body (including students), promoting public outreach via science fairs and exhibitions and multilingual scientific communication. We identified the need for funding opportunities for early career and established scientists that support these types of interdisciplinary interactions. Some efforts have already been made in the LAC region, such as RedLCC (by its acronymous in Spanish Red Latinoamericana de Cultura Científica), RedPop (Latin American and Caribbean Network for the Popularization of Science and Technology), the bilingual science communication portal Latin American Science and the Journal of Science and Communication (JCOM) América Latina^40^. In this sense, LAECESS is getting closer to the general public and is becoming a critical scientific communication tool by maintaining a webpage (https://laearlycareer.wixsite.com/latinamericaecess), posting multilingual posts on social media, organizing webinars, and currently working on an Earth Science divulgation podcast channel. Since LAECCES has no direct access to funds, this initiative focuses on highlighting the EC researchers’ work, sharing different calls and job offers as a strategy not only to share and divulge knowledge but also to expand the reach of young scientists in LAC. A potential new field for LAECCES is to develop funding requirements from research bodies to support the network.

### Early career involvement in decision-making

There is a need to improve interactions with decision-makers. In the LAC region, there are great examples of opportunities to develop science policy and diplomacy skills, such as the Inter-American Institute for Global Change Research projects and initiatives such as the Science, Technology, Policy (STeP) fellowship program or the Latin America and the Caribbean Open Science Forum (CILAC in Spanish)^41^. However, the participation of EC scientists is scarce due to the limited space in decision-making combined with a lack of science policy and diplomacy training. Closer collaboration among mid-careers scientists, late-careers scientists, policy-makers, and diplomats could solve this scarce participation as well as encourage industries, companies and governments to link young career scientists considering the climate challenges for the next 10 years^41^ 41. Governments, universities, and scientific institutions in LAC could adapt successful international programs to incorporate EC scientists into the science policy and science diplomacy areas. Two examples of these programs are the Colorado Science & Engineering Policy Fellowship (https://coscienceengineeringpolicyfellowship.com) and the Research Fellows at the European Commission (https://ec.europa.eu/jrc/en/working-with-us/jobs/vacancies/function-group-iv-researchers).

Additionally, the participation of EC scientists in town hall meetings, policy brief developments, and decision-making platforms is crucial to sharing their knowledge with society. Here scientific communication, especially on social media, become essential by providing EC scientists with a flexible, dynamic, and direct option to share scientific knowledge with the general public.

### Improved research funding opportunities

Research funding in the LAC region is needed for the technological and innovation development of the region, and in LAC, funding mainly comes from governments. Therefore, LAC-based funding opportunities are limited compared to other areas of the world where funding is expanded to private or independent research institutions. The collaboration with Europe has allowed for some advances, such as EURAXESS Latin America and the Caribbean (which links researchers in LAC countries with Europe) or the EU-CELAC platform (an information and communication site for funding agencies, universities, research centers, enterprises, and individuals interested in the bi-regional cooperation European Union). Additionally, LAC researchers are allowed to apply for Horizon Europe grants. However, these opportunities are not LAC-based, and progress into building LAC funding must be pursued. In this sense, the UNESCO-CEPAL, the Interamerican Development Bank, or Mercosul offer some funding opportunities for scientific research that are valuable but still scarce to fill the LAC research requirements.

The LAC early career scientists would greatly benefit from creating an open-access database or search engine with potential available funding opportunities at all levels, including individual funding, collaborative funding, private funding, training opportunities, and guidelines for successful applications. LAECESS is currently working on implementing such a project. To achieve this, collaboration among local and regional research networks to help gather this database and to build a search engine is essential. Additionally, an important asset would be the involvement of senior scientists to share examples of previous funding opportunities and collaborative projects in the LAC region that received international funds.

### Collaboration with other networks and stakeholders

There is a need to include a wide range of stakeholders in the knowledge production process. In this regard, engaging in transdisciplinary initiatives is beneficial for both the academic and non-academic worlds. This endeavour requires early career networks, such as LAECESS, to advocate for improved research programs open to contributions from practitioners, the private sector and communities.

LAECESS has collaborated with other early career and non-early career scientific networks to promote and organize seminars and is currently working to develop a podcast channel, webinars, and a resource database. An area of opportunity for LAECESS to increase the exposure of LAC research could be the collaboration with U.S.-based organizations with Hispanic and Latin-American branches. Examples are the American Meteorological Society Committee on Hispanic and Latinx Advancement (AMS CHALA), the Caribbean Institute for Meteorology and Hydrology, or the Society of Latinxs/Hispanics in Earth and Space Science (SOLESS). Some of these organizations have experience with collaborative efforts across academic institutions, scientific networks, and governmental agencies that could help LAECESS structure a path for establishing these types of connections in the future.

### Increase representation of underrepresented groups in LAC earth Sciences

As described in section 1.4, LAECESS recognizes the need to increase the representation of underrepresented groups in LAC. To that end, we are working on creating social media content to boost discussions on how to increase the number of women and other underrepresented groups in science, especially in Earth system sciences; this includes discussions on how to promote women’s access to seniority positions within their countries of origin.

## Final remarks

Tacking current environmental issues in LAC and the world requires joint international efforts and rethinking how scientific knowledge is fed into policymaking. Additionally, the inclusion of the whole society in the knowledge production process is vital to better understanding current problems and finding prompt and efficient solutions to them. The situation in the LAC region is of special importance due to current socio-economic conditions that prevent EC scientists from pursuing a career in the region. In this sense, the LAECESS network was created to promote regional networking, foster integrated and inter- and transdisciplinary science, organize soft skills courses and workshops and empower Latin American EC researchers. Yet, much work is still ahead of us. For better scientific and social development in the LAC region, we prioritize future steps to improve network sustainability over time, the need for a regional open-access information tool, the improvement of scientific communication, EC involvement in decision-making, and work towards enhanced research funding opportunities.

## Data Availability

No new data were created for this report.
